# Research enrichment: evaluation of structured research in the curriculum for dental medicine students as part of the vertical and horizontal integration of biomedical training and discovery

**DOI:** 10.1186/1472-6920-8-9

**Published:** 2008-02-19

**Authors:** Karl Kingsley, Susan O'Malley, Tanis Stewart, Katherine M Howard

**Affiliations:** 1 Department of Biomedical Sciences, School of Dental Medicine, University of Nevada, Las Vegas, USA; 2 Department of Information Technology, School of Dental Medicine, University of Nevada, Las Vegas, USA

## Abstract

**Background:**

Research programs within medical and dental schools are important vehicles for biomedical and clinical discovery, serving as effective teaching and learning tools by providing situations in which predoctoral students develop problem-solving and critical-thinking skills. Although research programs at many medical and dental schools are well-established, they may not be well integrated into the predoctoral curriculum to effectively support the learning objectives for their students.

**Methods:**

A series of structured seminars, incorporating faculty research, was designed for first-year dental students at the University of Nevada, Las Vegas, School of Dental Medicine to reinforce and support the concepts and skills taught in concurrent courses. A structured research enrichment period was also created to facilitate student engagement in active research using faculty and student curricular release time. Course evaluations and surveys were administered to gauge student perceptions of the curricular integration of research, the impact of these seminars on recruitment to the research program, and overall levels of student satisfaction with research enrichment.

**Results:**

The analysis of course surveys revealed that students perceived the research-containing seminars effectively illustrated concepts, were logically sequenced, and were well-integrated into their curriculum. In addition, analysis of surveys revealed that the Integration Seminar courses motivated students to engage in research enrichment. Finally, this analysis provided evidence that students were very satisfied with their overall learning experience during research enrichment.

**Conclusion:**

Curricular integration is one method of improving the teaching and learning of complicated and inter-related concepts, providing an opportunity to incorporate research training and objectives into traditionally separate didactic courses. Despite the benefits of curricular integration, finding the most appropriate points of integration, obtaining release time for curricular development and for research engagement, and funding predoctoral student research remain issues to be addressed in ways that reflect the character of the faculty and the goals of each institution.

## Background

Research is an important component of many predoctoral medical and dental education programs focused on training students as clinicians, educators, and researchers in evidence-based practices [1-3]. Biomedical and clinical research programs in these schools not only add to the body of knowledge regarding health and disease, but also serve as vehicles to train and mentor predoctoral students, motivating some students to pursue research and academic careers [4,5]. Other benefits to students include enhanced critical-thinking and problem-solving skills and increased biomedical perspectives [2].

One key to implementing effective research programs is the degree to which they can be successfully integrated into the curriculum. Medical and dental educators increasingly recognize that the traditional approach to teaching biomedical concepts and clinical training often lack both cohesiveness and an emphasis on research training and education [1,3,5]. To partially address these problems, many medical and dental schools now teach related biomedical, clinical, and behavioral concepts concurrently in an innovation known as horizontal integration. Horizontal integration contextualizes biomedical information using clinical illustrations and highlights inter-relationships among concepts [6,7]. A related curricular innovation, vertical integration, allows for the presentation of more complex cases, based specifically upon the structured acquisition and application of concepts from the preceding horizontally integrated coursework [8,9].

The expectation is that horizontal and vertical integration will result in a better understanding of important biomedical principles, improved retention of knowledge, and an enhanced ability for students to apply biomedical concepts in clinically appropriate settings [10,11]. One recent example of a vertically and horizontally integrated curriculum is found at the Marquette University School of Dentistry. Marquette significantly restructured its curriculum to minimize traditional discipline-based coursework while refocusing on interdisciplinary content that provides both horizontal and vertical integration of biomedical, behavioral, and clinical science concepts and topics [12]. Evidence demonstrates integrating new knowledge and technologies, traditionally isolated from the curriculum as faculty research, can enhance biomedical and clinical training for students [13]. New practices which promote and facilitate the integration of research into the curriculum are now beginning to develop and mature [14,15].

Marquette was one of the first dental schools to restructure its curriculum to incorporate a research emphasis. This research emphasis fosters appreciation of biomedical and clinical research and discovery through dedicated didactic time devoted to mentored scholarly research from various disciplines [12]. This curricular reorganization required the creation of flexible schedules to facilitate mentored research and scholarly experiences for the participating faculty and students. The result was a new model for curricular planning, development, implementation, and assessment specifically designed to foster student appreciation of research, discovery, and scholarly activity, in addition to influencing career development and applications to patient care [15].

The University of Nevada, Las Vegas, School of Dental Medicine (UNLV-SDM) has similarly defined its primary education goal as the implementation of "a vertical and horizontal integration of the Biomedical Sciences, Professional Studies, and Clinical Sciences curricula to ensure competent, contemporary oral healthcare providers" [16]. The UNLV-SDM has thus structured its curriculum to facilitate the seamless integration of the courses within the curriculum. The intent is that integration will foster a dental workforce possessing an informed and holistic approach to oral health care delivery [17]. Recognizing the value of a research program to dental students, the UNLV-SDM developed a structured program of research enrichment that is horizontally and vertically integrated into the curriculum.

This report describes the development and structure of the integrated research program at the UNLV-SDM, as well as internal evaluation measures and plans for future development of the program. In brief, participating research faculty first coordinated and presented research seminars in a first-year course, the Integration Seminar [18]. These research seminars were horizontally integrated with other key biomedical and clinical concept courses, both as a method of instruction and as a point of recruitment for the research program. Next, a structured research enrichment period was established in which participating faculty and students engaged in various research-related activities. Finally, this research was vertically integrated into the curriculum. Students who participated in research lectured in subsequent Integration Seminar courses, disseminating research findings and stimulating interest in potential student-driven research projects for subsequent enrichment periods.

Our assessment suggests the research enrichment program was both vertically and horizontally integrated into the curriculum at the UNLV-SDM. Course evaluations of the Integration Seminar demonstrated that first-year dental students overwhelmingly recognized that the research presentations were integrated and logically sequenced. Furthermore, an internal assessment of pre- and post-research enrichment surveys demonstrated that students were satisfied with their research enrichment experience.

## Methods

### Curriculum description

#### Integration Seminar

While the UNLV-SDM structured a predoctoral curriculum integrating the majority of courses, a first-year, two-semester course was designed and implemented to further facilitate both horizontal and vertical integration of basic and clinical sciences. Each first-year cohort of 75 dental students (DS1) takes two semesters of the Integration Seminar course, during the Fall and Spring semesters, receiving one credit per semester, graded on a Pass/Fail system determined by student participation and attendance. Course directors from integrated DS1 courses develop topics for each semester of the Integration Seminar, examining a central theme, issue, or contemporary health care problem that parallels and strengthens the course concepts addressed in concurrent DS1 courses [18].

#### Research enrichment

In addition to the standard predoctoral curriculum at UNLV-SDM, students may participate in Research Enrichment, a voluntary elective with a research emphasis. Research Enrichment is a period of curricular release time, between the DS1 Spring and Summer semesters, when students may pursue scholarly activities with a designated mentor. Selected research faculty are given protected time to plan, administer, teach, mentor, and evaluate structured research training for interested DS1 students.

More specifically, the basic format for each research enrichment period involves approximately one month of dedicated curriculum release time for the dental students and designated research faculty. Guidelines, goals, and expectations for performing research are distributed by the Associate Dean for Research prior to the beginning of this period and students are matched with an appropriate mentor based upon a combination of scientific interest and experience. The first week of this period involves general orientations, mandatory UNLV Environmental Health and Safety training, and specialized Biosafety Level-2 (BSL-2) training. The remaining weeks are spent developing and performing hypothesis-driven experiments, documenting research results, and subsequently analyzing these results for dissemination via publication or meeting presentation.

### Curricular implementation and assessment

#### Integration Seminar: incorporation of research

DS1 course instructors who provide evidence of integrated course materials and instruction between two or more departments or disciplines are selected to participate in the Integration Seminar. Participating faculty selected the concepts and topics for these seminars, including topics that integrated current, applied research under their direction. The pool of faculty for the years evaluated for this report included all faculty from Biomedical Sciences (7), Professional Studies (5) and Clinical Sciences (28 full-time, 32 part-time) departments, as well as other UNLV and external and student speakers.

Potential faculty mentors designed and presented seminars within the Integration Seminar course, incorporating their clinical, professional, or behavioral sciences-related research. Their research was directly correlated with the learning objectives for the seminar, and was presented to initiate and facilitate interactive discussions with students regarding relationships and concepts that pertained to concurrent DS1 course offerings, thus fostering horizontal integration. These seminars familiarized all DS1 students with faculty-directed research projects and served as a potential point of recruitment for interested students. Seminars with content demonstrating the integration of faculty research into the seminar objectives were then tallied and reported.

#### Integration Seminar: course evaluation and assessment

We collected qualitative course evaluation data over a three-year period, using the UNLV-SDM mandated, anonymous survey that was administered to each cohort (C1, C2, C3) of students at the end of each Integration Seminar course (N = 225) (Additional file 1). The survey consisted of 12 questions designed to gauge students' opinions of various aspects of the course, including the course format, course organization, and whether they believed integration was achieved within the course. Three questions, which most directly addressed student perception of research integration into the course, were analyzed. First, "the learning plan was smooth, sequenced, and logical" (Question 1). Second, "examples and illustrations were effective" (Question 2). Finally, "this course is integrated into the curriculum and not redundant" (Question 3). Each student evaluated the course by selecting one of the following choices for each statement about the course: "strongly agree" (SA), "agree" (A), "disagree" (D), "strongly disagree" (SD), or "not applicable" (NA).

#### Research enrichment recruitment

We collected additional qualitative data over the same three-year period, using an anonymous survey that was administered to students who subsequently participated in research enrichment (Additional file 2). The survey included 6 questions designed to gauge students' opinions about whether the Integration Seminar courses and the research-related presentations influenced their decision to engage in the research enrichment program. One particular question, Question 1, was designed to ascertain the degree to which Integration Seminar influenced their decision to participate in research enrichment. That question was: The Integration Seminar course motivated me to perform research during the Enrichment Period. Each student selected one of the following choices for each statement: "strongly agree" (SA), "agree" (A), "disagree" (D), "strongly disagree" (SD), or "not applicable" (NA).

#### Research enrichment goals

Dental students in this study performed biomedical laboratory research involving the following: i) environmental health and safety training, ii) specialized BSL-2 laboratory safety training, iii) individualized, hands-on mentoring in cell and molecular biology techniques, iv) training in the use of online literature databases, v) critical analysis and evaluation of peer-reviewed primary research articles, vi) statistical training specific to bias, confounding, study design, and hypothesis testing, vii) documentation of results, and viii) writing and editing of manuscripts for publication and abstracts for local or national meetings.

Upon completion of the research enrichment, it is expected that dental students are able to do each of the following:

• Understand and adhere to basic and advanced biomedical laboratory research safety protocols and standards;

• Understand and perform online literature searches and database utilization for biomedical and clinical research;

• Understand and discuss the protocols, limitations, and results of peer-reviewed biomedical and clinical research manuscripts;

• Design, execute, and evaluate research protocols using hypothesis testing;

• Accurately and descriptively summarize results for dissemination to biomedical and clinical researchers in abstract or manuscript form.

#### Research enrichment: evaluation and assessment

We collected qualitative evaluation data using an anonymous survey that was administered to students at the end of each research enrichment period and collected by an administrative assistant (N = 13) (Additional file 3). The survey consisted of 14 questions designed to gauge students' opinions and perceptions of their research experience. Many questions related to the organization and format of the research enrichment period; however, 5 questions more directly gauged students' overall perception of their experience, as well as their willingness to recommend it to other/future dental students. These questions were: (1) I found the Enrichment Period research option to be intellectually stimulating, (2) I am satisfied with my overall educational experience during the Enrichment Period, (3) If I had it to do over again, I would enroll in this Enrichment Period project, (4) I believe that other students would benefit from participation in this program, and (5) I would recommend the Enrichment Period research option to others. Each student completed the evaluation by selecting one of the following choices for each statement: "strongly agree" (SA), "agree" (A), "disagree" (D), "strongly disagree" (SD), or "not applicable" (NA).

#### Human subjects

Anonymous course evaluation data for the Integration Seminar courses (DEN7501 Integration Seminar I – Fall; DEN7502 Integration Seminar II – Spring) from 225 students in the three most recent UNLV-SDM cohorts were retrieved and provided in non-identifiable, summarized format by the Office of Student Affairs to prevent the disclosure, and ensure the confidentiality, of any potential personally identifiable private information.

Our protocol was approved by the Institutional Research Board (IRB), as an exemption to human subjects research under the Basic HHS Policy for Protection of Human Research Subjects, (46.101) Subpart A (b) regarding IRB Exemption for 1) research conducted in established educational settings involving normal educational practices, where the subjects cannot be identified or linked, directly or through identifiers.

## Results

### Integration Seminar: incorporation of research

In the first year (C1), 15 faculty members and speakers (6 Clinical, 4 Professional, 2 Biomedical, 3 external/other) created 20 seminars over the two semesters for the Integration Seminar, of which 2 seminars, or 10%, specifically integrated research (1 Clinical, 1 Biomedical). In the second year (C2), 18 faculty members and speakers (6 Clinical, 4 Professional, 3 Biomedical, 5 external/other) created 20 seminars for the Integration Seminar series, of which 9 seminars, or 45%, specifically integrated research (5 Biomedical, 2 Professional, 2 Clinical). In the third year (C3), 22 faculty members and speakers (6 Clinical, 4 Professional, 3 Biomedical, 9 external/other) created 22 seminars for these courses, of which 16, or 73%, specifically integrated research (6 Biomedical, 5 Professional, 5 Clinical).

### Integration Seminar: course evaluation and assessment

**Figure 1:**
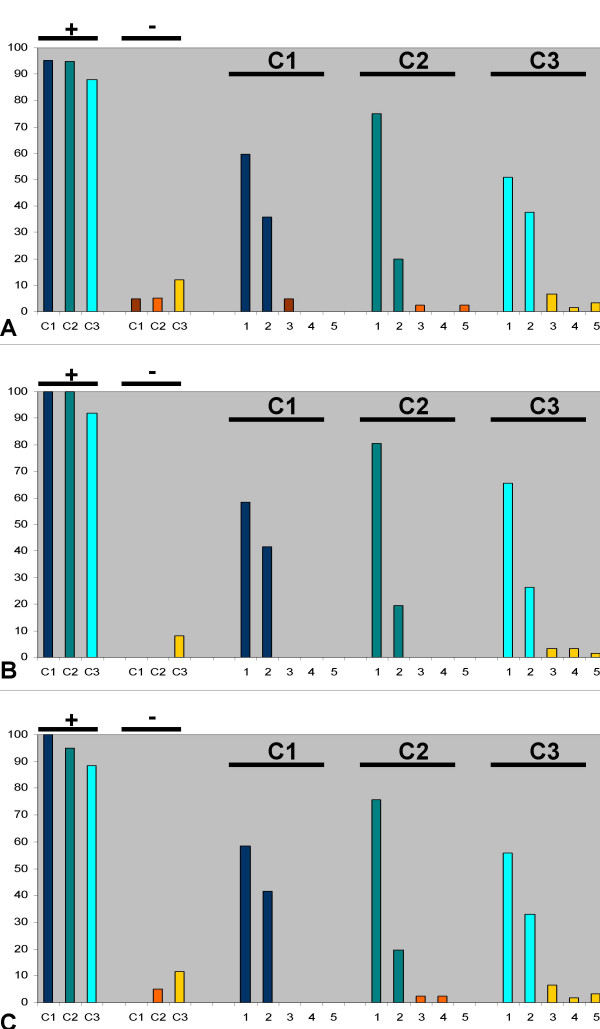
**Student perception of research integration**. Anonymous course evaluation survey data from three cohorts of dental students (C1, C2, C3) were analyzed (Total student pool = 225). Student responses ("strongly agree" = 1, "agree" = 2, "disagree" = 3, "strongly disagree" = 4, "not applicable" = 5) to three key questions regarding the Integration Seminar course are presented (Response rates: C1 = 80%, n = 120/150; C2 = 78%, n = 117/150; C3 = 80%, n = 120/150; Fall and Spring Integration Seminar course evaluations response combined, 75/cohort over two semesters = 150). Overwhelmingly, students from all cohorts had positive responses (+), either "strongly agree" or "agree" to these statements: A) "The learning plan was smooth, sequenced, and logical" (C1: 95.2%, C2: 95%, C3: 88%), B) "Examples and illustrations were effective" (C1: 100%, C2: 100%, C3: 92%), and C) "This course is integrated into the curriculum and not redundant" (C1: 100%, C2: 95.1%, C3: 88.53%).

The UNLV-SDM course evaluation was administered to each student cohort (75 students per cohort) at the end of both Fall and Spring semesters. The response rate among the three cohorts ranged from 78 to 80% (C1 = 80%, n = 120/150; C2 = 78%, n = 117/150; C3 = 80%, n = 120/150). Data were provided in non-identifiable, summarized format by the Office of Student Affairs, revealing only the response rate and percent of responses for each question. Analysis of qualitative course evaluation survey data revealed that the vast majority of students asserted that the components of this course were coherently sequenced, that cogent examples were used within the course, and that these components were well integrated into the curriculum (Figure 1). Specifically, between 88 and 100% of students in all three cohorts had positive responses, "strongly agree" (SA) or "agree" (A) to each question (Fig. 1A, B, C). Fewer than 10% of students had negative responses "disagree" (D) or "strongly disagree" (SD) to any question, with only a few responses indicating "not applicable" (NA).

More specifically, student responses to the first evaluated survey question, "The learning plan was smooth, sequenced, and logical", revealed that a significant proportion of students had positive perceptions regarding the sequence and logical order of the course components, which included the research presentations (Figure 1A). Almost all students in the first cohort (C1) had positive responses (95.2%), "strongly agree" plus "agree" to this question, which remained relatively constant at 95% and 88% among the subsequent cohorts (C2, C3, respectively), while 4.8%, 5%, and 12% had negative responses, "disagree", "strongly disagree" or "not applicable", respectively. The data for each individual cohort demonstrated that among the first cohort, C1, 59.5% "strongly agree", 35.7% "agree", 4.8% "disagree", while no students "strongly disagree" or indicated "not applicable". Data regarding student perception from the second and third cohorts, C2 and C3, revealed that 75% and 50.82% "strongly agree", 20% and 37.7% "agree", 2.5% and 6.56% "disagree", 0% and 1.64% "strongly disagree", and 2.5% and 3.28% marked "not applicable", respectively.

Student responses to the second evaluated survey question, "Examples and illustrations were effective", revealed that the overwhelming majority of students in each cohort had positive perceptions of the horizontally integrated clinical and research examples used in this course (Figure 1B). Virtually all students from each cohort (100% in C1, 100% in C2, 92% in C3) had positive responses. The data, organized by individual cohort, demonstrated that among students in C1 and C2, 58.5% and 80.5% "strongly agree", while 41.5% and 19.5% "agree", respectively. The data from C3 indicated that 65.57% "strongly agree", 26.23% "agree", 3.28% "disagree", 3.28% "strongly disagree", and 1.64% responded "not applicable".

Student responses to the third evaluated survey question, "This course is integrated into the curriculum and not redundant", once again demonstrated that most student from each cohort had positive perceptions of the horizontal integration of this course and the seminar material into the curriculum (Figure 1C). Nearly all students in each cohort (100% in C1, 95.1% in C2 and 88.53% in C3) had positive responses. Students in cohorts C1, C2, and C3 responded with "strongly agree" (58.5%, 75.6%, 55.74%), "agree" (41.5%, 19.5%, 32.79%), "disagree" (0%, 2.4%, 6.56%), "strongly disagree" (0%, 2.4%, 1.64%), or "not applicable" (0%, 0%, 3.28%), respectively.

### Research enrichment recruitment

In the first year (C1), 2 students participated in research enrichment. In the second year (C2), the number of students participating in research enrichment tripled to 6, and in the third year (C3), 5 students engaged in research enrichment. Seventy-seven percent (10/13) of dental students who participated in research enrichment from all three cohorts (C1, C2, C3) completed the recruitment survey (Table 1). The majority of students marked positive responses (80%), either "strongly agree" or "agree" to all statements regarding the influence of the Integration Seminar in motivating them to perform research during the enrichment period, while no students marked either "disagree" or "strongly disagree".

**Table 1:** *Influence of Integration Seminar on research enrichment recruitment

	Strongly Agree	Agree	Neutral	Disagree	Strongly Disagree
	C3 (N = 5) **() = C1+C2+C3 (N = 13)**				

1. The Integration Seminar (DEN7501/2) course motivated me to perform research during the Enrichment Period.	0.25 **(0.40)**	0.75 **(0.50)**	**(0.10)**		
2. Presentations from student (dental) researchers influenced my decision to perform research during the Enrichment Period.	0.50 **(0.40)**	0.25 **(0.40)**	0.25 **(0.20)**		
3. Presentations from faculty (dental) researchers influenced my decision to perform research during the Enrichment Period.	**(0.20)**	1.00 **(0.70)**	**(0.10)**		
4. Presentations from faculty (other colleges or departments) researchers influenced my decision to perform research during the Enrichment Period.	**(0.10)**	0.75 **(0.70)**	0.25 **(0.20)**		
5. I am interested in continuing my research project, to some degree, after the Enrichment Period ends.	1.00 **(1.00)**				
6. I am interested in presenting my research to DS1 students in the upcoming Integration Seminar.	0.75 **(0.70)**	0.25 **(0.30)**			

*presented as percentage of respondents

### Research enrichment goals

All of the students in each research enrichment cohort (C1, C2, C3) completed the environmental health and safety training, BSL-2 laboratory safety training, individual, hands-on mentoring in cell culture and molecular biology techniques, as well as training in the use of online literature databases. Furthermore, these students were allowed the opportunity to expand and develop their statistical competence and critical analysis and writing skills, as evidenced by their recent publication successes. Both students from the initial cohort (C1) have successfully co-authored manuscripts [19,20], as did all 6 students from the second cohort (C2) [21] and 2 students from the third cohort (C3) [20].

### Research enrichment: evaluation and assessment

The qualitative evaluation survey for the enrichment period was administered to all three cohorts of dental students who volunteered for the research enrichment period (Table 2). Analysis of the survey data for the first two cohorts (C1, C2) is reported with a completion rate of 75% (6/8); however, data for the third cohort were still being collected and were not available for incorporation into this report. In brief, all of the students who responded felt that they were intellectually stimulated, satisfied, and would recommend this research enrichment period option to other/future dental students. All students in the first two cohorts had positive responses, "strongly agree" (SA) or "agree" (A) to questions 1–5, while no students indicated negative responses "disagree" (D) or "strongly disagree" (SD).

**Table 2:** *Post-assessment of research enrichment period by dental students

	Strongly Agree	Agree	Disagree	Strongly Disagree	N/A
	C2 (N = 6) **() = C1+C2 (N = 8)**				

1. I found the Enrichment Period research option to be intellectually stimulating:*	0.80 **(0.66)**	0.20 **(0.34)**			
2. I am satisfied with my overall educational experience during the Enrichment Period:*	0.80 **(0.66)**	0.20 **(0.34)**			
3. If I had it to do over again, I would enroll in this Enrichment Period project:*	1.00 **(1.00)**				
4. I believe that other students would benefit from participation in this program:*	1.00 **(1.00)**				
5. I would recommend the Enrichment Period research option to others:*	0.80 **(0.83)**	0.20 **(0.17)**			
6. The laboratory personnel were accessible to me during the Enrichment Period:	1.00 **(1.00)**				
7. I experienced good working relationships with my mentor(s) during the Enrichment Period:	1.00 **(1.00)**				
8. I experienced good working relationships with the other student(s) during the Enrichment Period:	1.00 **(1.00)**				
9. I received an orientation to my research project at the beginning of the Enrichment Period:	1.00 **(0.80)**	**(0.20)**			
10. I received adequate safety training prior to beginning my research project:	1.00 **(0.80)**	**(0.20)**			
11. I received adequate scientific support and guidance to understand my research project:	0.80 **(0.83)**	0.20 **(0.17)**			
12. I felt that my questions regarding this project were answered to my satisfaction:	0.80 **(0.83)**	0.20 **(0.17)**			
13. I felt that my mentor(s) showed an interest/enthusiasm for my project:	1.00 **(1.00)**				
14. I believe my mentor(s) encouraged my participation and input for this project:	1.00 **(1.00)**				

*presented as percentage of respondents

More specifically, 75% of these students "strongly agree" that enrichment period was intellectually stimulating (Question 1) and were satisfied with their overall educational experience during the research enrichment period (Question 2). All students "strongly agree" that if they had it to do over again, they would enroll in this research enrichment period (Question 3). Furthermore, all students indicated that other dental students would benefit from participation in this program (Question 4), while 88% "strongly agree" that they would recommend the research enrichment period to others (Question 5).

In addition, several students volunteered to present their research data and results in the Integration Seminar during the following year and were counted as external/other speakers. Half of the participants from the first cohort, C1, presented to the following cohort, C2, as part of the Integration Seminar course and three-fourths of the second cohort, C2, presented their research results and data in the Integration Seminar course during the subsequent academic year. The most recent cohort, C3, will have the opportunity to present their research in the Integration Seminar course to the incoming cohort, C4, during the 2007–2008 academic year. Furthermore, a more detailed analysis of the survey data regarding the influence of the Integration Seminar to motivate dental students to perform research revealed that 80% of respondents had positive responses ("strongly agree" or "agree") to the statement that dental student presentations influenced their decision to enroll in the research enrichment period (Table 1, Question 2).

## Discussion

While many schools have established research programs, the concept of designing courses and curricula that facilitate the integration of research with curricular content for dental students remains a relatively new concept and one that is under continual development. The research enrichment period at the UNLV-SDM was designed to facilitate the vertical and horizontal integration of applied biomedical, professional, and clinical science research into the predoctoral dental school curriculum. As curriculum integration and biomedical discovery are both central to the overall mission of the UNLV-SDM, the development, implementation, and assessment of methods for fostering and integrating research is critical.

Although research rotations are compulsory in some medical and dental schools, both compulsory and voluntary predoctoral research programs face similar program challenges. These challenges usually involve the development and design of effective recruitment and retention methods to match prospective students with faculty mentors or the design and structure of research rotations themselves [2,5]. The Marquette University School of Dentistry provides not only an effective model, but also preliminary evidence to address these questions through a comprehensive faculty development initiative. This initiative included classes, seminars, and other training opportunities to develop instruction and teaching skills in research-oriented areas. In addition, Marquette established mentored research activities at local, national, and international sites, further expanding the range of learning and training opportunities not found at schools with traditional curricula [14].

This report is among the first to discuss not only the development and structure of a predoctoral research program, but also the horizontal and vertical integration of structured research into specific courses in the dental curriculum. Similar to the results at Marquette, the initial results of this study are also quite promising. Our efforts to incorporate faculty research into the Integration Seminar course resulted in their increase from 10% to 73% over three years. More importantly, student feedback indicated that students widely perceived this directed research as logical, effective, and integrated. It is hoped that replacing previously non-integrated instruction with integrated, faculty-driven research instruction and materials will result in improved understanding of underlying principles, higher retention of foundation concepts, and enhanced critical evaluation in the appropriate clinical settings.

In addition, the number of students participating in the nascent research programs at UNLV-SDM over the three years of the study has increased. One possible explanation for the increase is the growing number of research presentations incorporated into the Integration Seminar. More intriguing is the relationship between the number of student presentations (vertical integration) and the number of students recruited. More specifically, although greater than 80% of students cited the Integration Seminar as a motivational influence on their decision to participate in research enrichment, twice as many students "strongly agree" that they were more influenced by the presentation of previous student researcher participants than by faculty presentations (Table 1). Finally, it is important to note that the publication of student results in peer-reviewed journals may also be a factor in recruitment, as students from C1 and C2 cohorts have published their findings in cancer-specific journals [19-21].

Although this report describes the design and implementation of a research enrichment program for predoctoral dental students and the process and mechanism for integrating this research into components of the dental school curriculum, there are several limitations of this study. First, and most importantly, UNLV-SDM is a new program and has the benefits and problems associated with recently established schools. As a benefit, the curriculum and many of the faculty are new and were specifically recruited for their willingness to implement new curricular innovations, such as integration. Two challenges have been the limited number of faculty with established research programs and the limited amount of funds available for new, tenure-track faculty to support student research. Despite the limitations and challenges, the results of this report may be of value to other dental, medical, or professional schools with a similar goal of incorporating research and achieving curriculum integration.

## Conclusion

Medical and dental education curricula are continually developing by incorporating advancements, such as horizontal and vertical integration, to address the contemporary needs of their students. This study provides an example of how current research by faculty and predoctoral students can be incorporated in an organized and structured manner into the curriculum. In addition, several areas of future research and potential improvement were identified during the process of analyzing these data, which will be the subject of future studies. First is the separation of the Integration Seminar series into two courses, one as an introductory biomedical, professional, and clinical integration seminar series and the second as an applied, research-specific integration series focused primarily on student research and participation, and more specifically on dissemination of their results to incoming dental school cohorts. The second is evaluating the relative benefits of research participation on recruitment of future faculty members and in the selection of post-graduate specialty training.

## Competing interests

The author(s) declare that they have no competing interests.

## Authors' contributions

KK was responsible for design of the concept and for integrating research enrichment into the Integration Seminar courses. KK, SO, and KH developed the research enrichment goals and objectives and directed the research enrichment. SO and TS developed and coordinated the survey methods, data collection, and data analysis. All authors have read and approved the final version of this manuscript.

## Pre-publication history

The pre-publication history for this paper can be accessed here:



## Supplementary Material

Additional File 1 **UNLV-SDM course evaluation**. Anonymous survey administered to students at the completion of each Integration Seminar course. Click here for file

Additional File 2 **UNLV-SDM Enrichment Period recruitment survey**. Anonymous student survey designed to identify the factors that most impacted their decision to participate in research enrichment. Click here for file

Additional File 3 **UNLV-SDM Post-Enrichment Period survey instrument**. Anonymous surveys designed to gauge students' opinions of their research experience during the enrichment period. Click here for file
