# Supplementary material for: Research enrichment: evaluation of structured research in the curriculum for dental medicine students as part of the vertical and horizontal integration of biomedical training and discovery
Source: BMC Med Educ. 2008 Feb 19;8:9. doi: 10.1186/1472-6920-8-9 (PMC2267175; doi:10.1186/1472-6920-8-9)
Supplement: Additional File 1 — UNLV-SDM course evaluation. Anonymous survey administered to students at the completion of each Integration Seminar course. [file 1472-6920-8-9-S1.doc]

**Additional file 1**

**UNLV-SDM Course Evaluation**

**1. The learning plan was smooth, sequenced and logical.***

Strongly Agree Agree Disagree Strongly Disagree NA

**2. Examples and illustrations were effective.***

Strongly Agree Agree Disagree Strongly Disagree NA

**3. This course is integrated into the curriculum and not redundant.***

Strongly Agree Agree Disagree Strongly Disagree NA

4. Instructional materials were well-prepared and well organized.

Strongly Agree Agree Disagree Strongly Disagree NA

5. Materials, projects and evaluations were explained in a logical way.

Strongly Agree Agree Disagree Strongly Disagree NA

6. Directions were given clearly and concisely so that students could carry out assignments.

Strongly Agree Agree Disagree Strongly Disagree NA

7. Material was presented at student’s level of comprehension.

Strongly Agree Agree Disagree Strongly Disagree NA

8. Evaluations were fair, impartial, and respectful to students.

Strongly Agree Agree Disagree Strongly Disagree NA

9. The requirements and competencies were clearly stated.

Strongly Agree Agree Disagree Strongly Disagree NA

10. The length of the class sessions is appropriate.

Strongly Agree Agree Disagree Strongly Disagree NA

11. The number of instructors is appropriate.

Strongly Agree Agree Disagree Strongly Disagree NA

12. The course prepared you for the competency exam.

Strongly Agree Agree Disagree Strongly Disagree NA
