# Supplementary material for: Research enrichment: evaluation of structured research in the curriculum for dental medicine students as part of the vertical and horizontal integration of biomedical training and discovery
Source: BMC Med Educ. 2008 Feb 19;8:9. doi: 10.1186/1472-6920-8-9 (PMC2267175; doi:10.1186/1472-6920-8-9)
Supplement: Additional File 2 — UNLV-SDM Enrichment Period recruitment survey. Anonymous student survey designed to identify the factors that most impacted their decision to participate in research enrichment. [file 1472-6920-8-9-S2.doc]

**Additional file 2**

**UNLV School of Dental Medicine**

DEN7505 Enrichment period recruitment survey

**Instructions**: Please mark the box corresponding to your response to each question where appropriate. Unless otherwise instructed, only mark one response per question.

We are asking you to provide us with your candid responses for the Enrichment Period (DEN7505), and the potential influences that precipitated in your decision to perform research.

Please indicate the extent to which you agree or disagree with the following statements:

|  | Strongly  Agree | Agree | Neutral | Disagree | Strongly  Disagree |
| --- | --- | --- | --- | --- | --- |
| 1. The Integration Seminar (DEN7501/2) course motivated me to perform research during the Enrichment Period. |  |  |  |  |  |
| 2. Presentations from student (dental) researchers influenced my decision to perform research during the Enrichment Period. |  |  |  |  |  |
| 3. Presentations from faculty (dental) researchers influenced my decision to perform research during the Enrichment Period. |  |  |  |  |  |
| 4. Presentations from faculty (other colleges or departments) researchers influenced my decision to perform research during the Enrichment Period. |  |  |  |  |  |
| 5. I am interested in continuing my research project, to some degree, after the Enrichment Period ends. |  |  |  |  |  |
| 6. I am interested in presenting my research to DS1 students in the upcoming Integration Seminar. |  |  |  |  |  |
