# Supplementary material for: Research enrichment: evaluation of structured research in the curriculum for dental medicine students as part of the vertical and horizontal integration of biomedical training and discovery
Source: BMC Med Educ. 2008 Feb 19;8:9. doi: 10.1186/1472-6920-8-9 (PMC2267175; doi:10.1186/1472-6920-8-9)
Supplement: Additional File 3 — UNLV-SDM Post-Enrichment Period survey instrument. Anonymous surveys designed to gauge students' opinions of their research experience during the enrichment period. [file 1472-6920-8-9-S3.doc]

**Additional file 3**

**UNLV School of Dental Medicine**

DEN7505 Post-Enrichment Period Survey Instrument

**Instructions**: Please mark the box corresponding to your response to each question where appropriate. Unless otherwise instructed, only mark one response per question.

We are asking you to provide us with a candid assessment of your participation in this short academic Enrichment Period (DEN7505), the impact on your academic career, and your recommendations for improvement.

Please indicate the extent to which you agree or disagree with the following statements:

|  | Strongly  Agree | Agree | Disagree | Strongly  Disagree | N/A |
| --- | --- | --- | --- | --- | --- |
| **1. I found the Enrichment Period research option to be intellectually stimulating:*** |  |  |  |  |  |
| **2. I am satisfied with my overall educational experience during the Enrichment Period:*** |  |  |  |  |  |
| **3. If I had it to do over again, I would enroll in this Enrichment Period project:*** |  |  |  |  |  |
| **4. I believe that other students would benefit from participation in this program:*** |  |  |  |  |  |
| **5. I would recommend the Enrichment Period research option to others:*** |  |  |  |  |  |
| 6. The laboratory personnel were accessible to me during the Enrichment Period: |  |  |  |  |  |
| 7. I experienced good working relationships with my mentor(s) during the Enrichment Period: |  |  |  |  |  |
| 8. I experienced good working relationships with the other student(s) during the Enrichment Period: |  |  |  |  |  |
| 9. I received an orientation to my research project at the beginning of the Enrichment Period: |  |  |  |  |  |
| 10. I received adequate safety training prior to beginning my research project: |  |  |  |  |  |
| 11. I received adequate scientific support and guidance to understand my research project: |  |  |  |  |  |
| 12. I felt that my questions regarding this project were answered to my satisfaction: |  |  |  |  |  |
| 13. I felt that my mentor(s) showed an interest/enthusiasm for my project: |  |  |  |  |  |
| 14. I believe my mentor(s) encouraged my participation and input for this project: |  |  |  |  |  |
